# Muramyl Endopeptidase Spr Contributes to Intrinsic Vancomycin Resistance in *Salmonella enterica* Serovar Typhimurium

**DOI:** 10.3389/fmicb.2018.02941

**Published:** 2018-12-14

**Authors:** Kim Vestö, Douglas L. Huseby, Iina Snygg, Helen Wang, Diarmaid Hughes, Mikael Rhen

**Affiliations:** ^1^Department of Microbiology, Tumor and Cell Biology, Karolinska Institutet, Stockholm, Sweden; ^2^Department of Medical Biochemistry and Microbiology, Uppsala University, Uppsala, Sweden; ^3^Laboratory for Molecular Infection Medicine Sweden (MIMS), Department of Molecular Biology, Umeå Centre for Microbial Research (UCMR), Umeå University, Umeå, Sweden

**Keywords:** vancomycin, antibiotic resistance, Spr, MepS, YebA, MepM, Tsp, Prc

## Abstract

The impermeability barrier provided by the outer membrane of enteric bacteria, a feature lacking in Gram-positive bacteria, plays a major role in maintaining resistance to numerous antimicrobial compounds and antibiotics. Here we demonstrate that mutational inactivation of *spr*, coding for a muramyl endopeptidase, significantly sensitizes *Salmonella enterica* serovar Typhimurium to vancomycin without any accompanying apparent growth defect or outer membrane destabilization. A similar phenotype was not achieved by deleting the genes coding for muramyl endopeptidases MepA, PbpG, NlpC, YedA, or YhdO. The *spr* mutant showed increased autolytic behavior in response to not only vancomycin, but also to penicillin G, an antibiotic for which the mutant displayed a wild-type MIC. A screen for suppressor mutations of the *spr* mutant phenotype revealed that deletion of *tsp* (*prc*), encoding a periplasmic carboxypeptidase involved in processing Spr and PBP3, restored intrinsic resistance to vancomycin and reversed the autolytic phenotype of the *spr* mutant. Our data suggest that Spr contributes to intrinsic antibiotic resistance in *S*. Typhimurium without directly affecting the outer membrane permeability barrier. Furthermore, our data suggests that compounds targeting specific cell wall endopeptidases might have the potential to expand the activity spectrum of traditional Gram-positive antibiotics.

## Introduction

Peptidoglycan (murein) constitutes a main component of the bacterial cell wall. It is composed of repeated *N*-acetylglucosamine (GlcNAc) and *N*-acetylmuramic acid (MurNAc) disaccharide units, cross-linked by peptide bridges. The synthesis of this mesh is the target of several classes of antibiotics, such as the β-lactams and glycopeptides. Peptidoglycan functions to maintain bacterial shape, septum formation at the point of cell division, and cell integrity upon internal turgor stress. To facilitate changes in size and shape during growth, bacteria need enzymes that can assemble and disassemble peptidoglycan. The process of re-shaping peptidoglycan involves the concerted activities of periplasmic amidases, endopeptidases, glycosylases and transpeptidases (penicillin-binding proteins, PBPs) (Sauvage et al., 2008). While the PBPs have the important function of catalyzing the formation of interpeptide bridges between overlapping GlcNAc-MurNAc polymers, the murein endopeptidases are tasked with cleaving interpeptide bridges to facilitate the incorporation of new GlcNAC-MurNAc polymers into the growing peptidoglycan mesh. The importance of correctly balancing these opposing activities is illustrated by the fact that blocking PBP activity with β-lactam antibiotics results in autolysis in *Escherichia coli* (Prestidge and Pardee, 1957).

The outer membrane of Gram-negative enteric bacteria, due to its relative impermeability, provides an intrinsic resistance barrier against many large compounds, including the antibiotics erythromycin (Nikaido and Vaara, 1985; Delcour, 2009), novobiocin (Anderle et al., 2008), rifampicin and vancomycin (Weeks et al., 2010; Krishnamoorthy et al., 2013). Furthermore, the increasing frequency of clinical bacterial isolates producing extended-spectrum β-lactamases is limiting the effectiveness of antibiotics that target cell wall synthesis amongst Gram-negative species (Coque et al., 2008). The search for new antibiotics to treat Gram-negative bacterial infections would be advanced by a better understanding of bacterial cell wall homeostasis at the level of peptidoglycan. Because it is a genetically amenable bacterium, *E. coli* has been the main focus for studies on the activities of cell wall-modulating enzymes. From these studies a consensus has emerged that, apart from PBP2 and PBP3 (Botta and Park, 1981), each of the glycolytic, endopeptic hydrolases and PBPs are individually dispensable for bacterial viability. Accordingly, inactivation of any one (or sometimes more than one) of the genes encoding these enzymes [PBP4: (Matsuhashi et al., 1977; Denome et al., 1999; Meberg et al., 2004), PBP5: (Matsuhashi et al., 1979; Nishimura et al., 1980; Spratt, 1980; Denome et al., 1999), PBP6: (Broome-Smith and Spratt, 1982; Denome et al., 1999), PBP6b: (Baquero et al., 1996), PBP7/PBP8: (Henderson et al., 1995; Denome et al., 1999), reviewed in: (van Heijenoort, 2011)] does not prevent bacterial growth under laboratory conditions. While this might imply a high degree of functional redundancy, it does not exclude the possibility that some or all of these enzymes may have unique functions under other more specific conditions.

A recent study (Singh et al., 2012) confirmed muramyl endopeptidase activity for three additional *E. coli* proteins; Spr, YebA and YdhO, renamed in *E. coli* to MepS, MepM, and MepH (Singh et al., 2015). More specifically, the study presented data implying that Spr or YebA might represent endopeptidases with less redundant functions, since it was feasible to construct an Δ*spr*Δ*yebA* mutant only in an *E. coli* strain genetically complemented with *spr* (*mepS*) (Singh et al., 2012).

*Salmonella enterica* serovar Typhimurium (*S.* Typhimurium) is a Gram-negative enterobacterium with an increasing antibiotic resistance development in its genus (Angelo et al., 2016; Hong et al., 2016; Klemm et al., 2018). As the *S*. Typhimurium genome includes genes with high sequence similarity to the *mepS, mepM*, and *mepH* genes of *E. coli*, and given the potential importance of *mepS* and *mepM* for viability of *E. coli* (Singh et al., 2012), we studied the phenotypes of *S.* Typhimurium mutants in which these genes were deleted, either singly or in combination. We characterized Δ*spr*, Δ*yebA*, and Δ*ydhO* mutants in terms of their growth and susceptibility profiles against antimicrobials, and in addition Δ*spr* mutant for autolytic behavior. Our findings highlight Spr as a possible new target for antibacterial treatment in order to sensitize *Salmonella* against Gram-positive-specific antibiotics.

## Materials and Methods

### Bacterial Strains

Mutants were constructed in the *Salmonella enterica* serovar Typhimurium SR-11 background (Sukupolvi et al., 1997), and are listed in Table 1. The *S*. Typhimurium strains LB5010 (Bullas and Ryu, 1983), ATCC 14028 and *E. coli* Top10, TG1 were used as intermediary hosts during mutant constructions or cloning. Furthermore, *S*. Typhimurium strains ATCC 14028s and SL1344 were also used to host a Δ*spr* mutation. The pACYC184-derived plasmid coding for β-galactosidase was available from a previous work (Taira et al., 1991).

**Table 1:** Strains used in study.

KV82	*Salmonella enterica* serovar Typhimurium strain SR-11 wild-type
KV141	*Salmonella enterica* serovar Typhimurium strain 14028 wild-type
KV110	*Salmonella enterica* serovar Typhimurium strain LB5010 wild-type
KV199	*Salmonella enterica* serovar Typhimurium strain SL1344 wild-type
KV154	LB5010:pSIM6
KV145	14028:pSIM5-tet
KV224	SR-11:pSIM6
KV244	SR-11 Δ*spr*
KV268	SR-11 Δ*yebA*
KV407	SR-11 Δ*ydhO*
KV267	SR-11 Δ*spr*Δ*yebA*
KV408	SR-11 Δ*spr*Δ*ydhO*
KV409	SR-11 Δ*yebA*Δ*ydhO*
KV240	SR-11 Δ*spr*::*cat*
KV255	SR-11 Δ*yebA*::*cat*
KV403	SR-11 Δ*ydhO*::*neo*
KV259	SR-11 Δ*spr*Δ*yebA*::*cat*
KV404	SR-11 Δ*spr*Δ*ydhO*::*neo*
KV405	SR-11 Δ*yebA*Δ*ydhO*::*neo*
KV235	LB5010 Δ*spr*::*cat*
KV249	14028 Δ*yebA*::*cat*
KV397	LB5010 Δ*ydhO*::*neo*
KV386	SR-11 Δ*tsp*
KV387	SR-11 Δ*spr*Δ*tsp*
KV388	SR-11 Δ*spr*Δ*yebA*Δ*tsp*
KV374	SR-11 Δ*tsp*::*cat*
KV378	SR-11 Δ*spr*Δ*tsp*::*cat*
KV382	SR-11 Δ*spr*Δ*yebA*Δ*tsp*::*cat*
KV370	LB5010 Δ*tsp*::*cat*
KV83	SR-11:pBAD30
KV247	SR-11 Δ*spr*:pBAD30
KV295	SR-11 Δ*spr*:pBAD30::*spr*
KV308	SR-11 Δ*spr*:pBAD30::*sprC70S*
KV322	SR-11 Δ*spr*Δ*yebA*:pBAD30
KV305	SR-11 Δ*spr*Δ*yebA*:pBAD30::*spr*
KV289	LB5010:pBAD30::*spr*
KV302	LB5010:pBAD30::*sprC70S*
KV278	*Escherichia coli* TG1:pBAD30*spr*
KV299	*Escherichia coli* Top10:pBAD30*sprC70S*
KV418	SR-11 Δ*spr*Δ*tsp*:pBAD30
KV416	SR-11 Δ*spr*Δ*tsp*:pBAD30::*tsp*
KV419	SR-11 Δ*spr*Δ*yebA*Δ*tsp*:pBAD30
KV417	SR-11 Δ*spr*Δ*yebA*Δ*tsp*:pBAD30::*tsp*
KV413	LB5010:pBAD30::*tsp*
KV411	*Escherichia coli* Top10:pBAD30::*tsp*
KV424	SR-11 Δ*rfaC*::*cat*
KV427	SR-11 Δ*rfaG*::*neo*
KV428	SR-11 Δ*rfaP:*:*cat*
KV433	SR-11 Δ*mepA*::*neo*
KV434	SR-11 Δ*pbpG*::*neo*
KV435	SR-11 Δ*nlpC*::*neo*
KV430	LB5010 Δ*mepA*::*neo*
KV431	LB5010 Δ*pbpG*::*neo*
KV432	LB5010 Δ*nlpC*::*neo*
KV392	SR-11 Δ*proQ*
KV393	SR-11 Δ*spr*Δ*proQ*
KV394	SR-11 Δ*spr*Δ*yebA*Δ*proQ*
KV395	SR-11 Δ*htpX*
KV396	SR-11 Δ*spr*Δ*htpX*
KV376	SR-11 Δ*proQ*::*cat*
KV380	SR-11 Δ*spr*Δ*proQ*::*cat*
KV384	SR-11 Δ*spr*Δ*yebA*Δ*proQ*::*cat*
KV377	SR-11 Δ*htpX*::*cat*
KV381	SR-11 Δ*spr*Δ*htpX*::*cat*
KV385	SR-11 Δ*spr*Δ*yebA*Δ*htpX*::*cat*
KV372	LB5010 Δ*proQ*::*cat*
KV373	LB5010 Δ*htpX*::*cat*
KV436	SR-11 Δ*nlpI*::*neo*
KV437	SR-11 Δ*spr*Δ*nlpI*::*neo*
KV438	SR-11 Δ*spr*Δ*yebA*Δ*nlpI*::*neo*
KV441	SR-11:pKTH3088
KV442	SR-11 Δ*spr*:pKTH3088
KV445	SR-11 Δ*spr*Δ*yebA*:pKTH3088
KV446	SR-11 Δ*spr*Δ*tsp*:pKTH3088
KV448	SR-11 Δ*spr*Δ*yebA*Δ*tsp*:pKTH3088
KV449	14028 Δ*spr*::*cat*
KV450	SL1344 Δ*spr*::*cat*
FIA1500	SR-11 Δ*spr*Δ*yebA* + Δ nt 1943335–1949114 (suppressor mutant)
FIA1501	SR-11 Δ*spr*Δ*yebA* + Δ nt 1943335–1949114 (suppressor mutant)
FIA1502	SR-11 Δ*spr*Δ*yebA* + Δ nt 1939839–1948327 (suppressor mutant)
FIA1503	SR-11 Δ*spr*Δ*yebA* + Δ nt 1923856–1964581 (suppressor mutant)
FIA1504	SR-11 Δ*spr*Δ*yebA* + Δ nt 1940175–1942805 (suppressor mutant)
FIA1505	SR-11 Δ*spr*Δ*yebA* + Δ nt 1939318–1964299 (suppressor mutant)
FIA1506	SR-11 Δ*spr*Δ*yebA* + Δ nt 1923856–1964581 (suppressor mutant)
FIA1507	SR-11 Δ*spr*Δ*yebA* + Δ nt 1923856–1964581 (suppressor mutant)
FIA1508	SR-11 Δ*spr*Δ*yebA* + Δ nt 1926281–1948787 (suppressor mutant)
FIA1509	SR-11 Δ*spr*Δ*yebA* + Δ nt 1923856–1964581 (suppressor mutant)
FIA1510	SR-11 Δ*spr*Δ*yebA* + *tsp* T479R (suppressor mutant)
FIA1511	SR-11 Δ*spr*Δ*yebA* + Δ nt 1923856–1964581 (suppressor mutant)
FIA1512	SR-11 Δ*spr*Δ*yebA* + Δ nt 1943335–1949114 (suppressor mutant)
FIA1513	SR-11 Δ*spr*Δ*yebA* + Δ nt 1943335–1949114 (suppressor mutant)

### Media and Growth Conditions

Growth media included tryptone and yeast extract (Sigma-Aldrich, 10 g/l respective 5 g/l) with 10 g/l of NaCl (LB medium), or without NaCl (TY medium). Cultures were incubated at 37°C unless otherwise stated. When needed, antibiotics were added to the growth media at the following concentrations: ampicillin 100 μg/ml; chloramphenicol 25 μg/ml; kanamycin 50 μg/ml; tetracycline 10 μg/ml. All antibiotics were purchased from Sigma-Aldrich (Sweden).

For determining growth curves, bacteria were incubated overnight in 2 ml LB at 220 rpm and 37°C. The next day, 150 μl of the culture was mixed with 850 μl of PBS and the OD_600 nm_ measured. The bacteria were then adjusted to an OD_600 nm_ of 0.25 (Ultrospec 1000, Pharmacia Biotech). Bacteria were further diluted 1:25 in either LB or TY broth resulting in a final OD_600 nm_ of 0.01. 400 μl of this bacterial suspension was then loaded into wells in a Honeycomb Bioscreen plate (OY Growth Curves AB Ltd., Helsinki, Finland) in three technical replicates. Uninoculated media was used as a negative growth control. The Bioscreen C plate reader (OY Growth Curves AB Ltd.) was set to an OD of 600 nm and optical density measurement was taken every 15 min with 5 s of agitation before every measurement, up until 24 h.

### PCR and Oligonucleotides

Polymerase chain reaction (PCR) was performed using an Eppendorf Mastercycler Personal. Oligonucleotides were designed using the genome of *S.* Typhimurium LT2 as reference (McClelland et al., 2001). For the generation of the inserts for gene deletions, the PCR was performed using Phusion High-Fidelity PCR master mix with HF buffer (New England Biolabs, United States). The cycling conditions were as following: 98°C for 1 min and 30 cycles of 98°C for 15 s, 54.5°C for 10 s, 72°C for 40 s, and 72°C for 2 min. The oligonucleotides were ordered from Sigma-Aldrich and specified in Table 2.

**Table 2:** Oligonucleotides for construction of strains and diagnostic PCR.

FSprrec	CGATATTTATCGTTAAGGACTTCAAGGGAAAACAAACAAC GTGTAGGCTGGAGCTGCTTC
Rsprrec	TCTCATCAGGTAAGCCAAGGGAGGTGCTGCCTGATGAAGA CATATGAATATCCTCCTTAG
Fspr(c)	GAA TTG TCT CAA GCT GTG CAG G
Rspr(c)	ATT CGG CAA AAC GGG TTC AG
FYebArec	TGCGAGCTGCCTGAAAGGAGATTAACGAGGAAGTGAATAC GTGTAGGCTGGAGCTGCTTC
RYebArec	AGC CGG CAC ACA TCG CGT ACC GGC TCT GTC AGC GCA TTT G CATATGAATATCCTCCTTAG
FYebA(c)	TTA GCC AAC CAG TAT GCG AGC
RYebA(c)	GTA GCG ACG TCT GCG TCT C
FYdhOrec	GTAGATTAGAATTATCAGGTTTTGTAAATCATACGCAGGC GTGTAGGCTGGAGCTGCTTC
RYdhOrec	AAG AAG AAG TTA TCC TGT CGT TAA ACG ACA GGA TAA AAT A CATATGAATATCCTCCTTAG
FYdhO(c)2	CTG AAG CCT GTC ATT GTA ACG G
RYdhO(c)2	CGA TCT CTT CCA GCG ATT TGC
FTsprec	ATGTCTTTGATTGTACGCGCAGAACACCTGGTGTTCTGAA GTGTAGGCTGGAGCTGCTTC
RTsprec	TTA AAA AAA AAC AGG CAC AAT TTT TTG TGC CTG TTT AGC GCATATGAATATCCTCCTTAG
FTsp(c)	TCA CCA AAG ATG GTG TCC GT
RTsp(c)	TAT CCT GAC GAC TTC TGC GC
FRfaCrec	GCAGCGGGTTCTGGAAGAGCTTCATTCGCTGTTGTCGGAA GTGTAGGCTGGAGCTGCTTC
RRfaCrec	TCT TTT CTC CAC AAT AGG TTT GGG ATG AGA CAG AGT CTC T CATATGAATATCCTCCTTAG
FRfaC(c)	AAG TGC GTA AAG GTG ATA CGG
RRfaC(c)	CGC TTT ATT CCA GAT CGG CTT
FRfaPrec	GATTTATACAGCTTACCGGAGAAGGCCGCGGATATTATTA GTGTAGGCTGGAGCTGCTTC
RRfaPrec	CTC ACT CAT AAA TTA CTC ACT GAG TGC ATA ATT ATT ATA A CATATGAATATCCTCCTTAG
FRfaP(c)	ACA CAG CCT TCC TTA CGC AA
RRfaP(c)	GCC AGC AGG TGT GGC AAT ATA
FRfaGrec	GACGGAAAAAATGCTGCCGCATGAGGCACGCACCATAGAT GTGTAGGCTGGAGCTGCTTC
RRfaGrec	ATC TTT ACC GCG CCA TAG TGT GGT TAA CGG CGC TTT CAG CCATATGAATATCCTCCTTAG
FRfaG(c)	TAC CTT TCC GTT ATT CCG GCT G
RRfaG(c)	GTC TCC AGC TCT CTG AAC AC
FMepArec	ATCGGGCACAGAATGCGGATGTAAAGACAGAGATTCCACG GTGTAGGCTGGAGCTGCTTC
RMepArec	AGC AGC GGG GAG ACC ATA AAC AGA TCA TAA AAA TTG TCC A CATATGAATATCCTCCTTAG
FMepA(c)	AGT GCC GAT CGC AGA AG
RMepA(c)	AAA TCC TGC CAG TAC GGC
FNlpCrec	CAGGTAATTTCGACGCTAAATTAATACCAAAATAAAAACA GTGTAGGCTGGAGCTGCTTC
RNlpCrec	ATG TTA AAA ATA GAC TAT AAA ATT TAT ATC GTC TGC GAG GCATATGAATATCCTCCTTAG
FNlpC(c)	CGT CGA GGG GCA TCC AAT
RNlpC(c)	AGT TCA ACC GGC GAT ATG TT
FPbpGrec	AGCTCAGGCGGTGTGCGTTACGACGCGCGTGAATCATTAT GTGTAGGCTGGAGCTGCTTC
RPbpGrec	TGA AGC CCG GCG GCG CGA TGC CTG CCG GGC CTG CGG CGA C CATATGAATATCCTCCTTAG
FPbpG(c)	TTC TGT AGC GGC AAC GCT
RPbpG(c)	GCG GAA ATT CTG GCA GGA A
FsprSacI2	CATGAGCTCAGGAGGA CAA CAT GGT CAA ATC TCA ACC G
RsprHindIII	CATGAAGCTT TTA ACT GCG GCT CAG AAC TC
FSprC68S (Primer for making C70S)	GCAGCACTAAGAAAGGCGTCGACTCTTCC AGC TTT GTA CAG CGC ACC TTC
RSprC68S (Primer for making C70S)	GAA GGT GCG CTG TAC AAA GCT GGA AGA GTC GAC GCC TTT CTT AGT GCT GC
pBAD30Forward	TTA GCG GAT CCT ACC TGA CG
FTspEcoRI	CATGAATTCAGGAGGA TGT TCT GAA ACG GAG GCC A
RTspHindIII	CATGAAGCTT TTA CTT ATT GGC TGC CGC CT
FProQrec	CTGTTCATGCCTGCTGCTTGTTGGCTACGTCCGTTGTAAT GTGTAGGCTGGAGCTGCTTC
RProQrec	AAG CCT AAA AAA AGT GTT CAT GCC AGG CCT GGC CTC CGT TCATATGAATATCCTCCTTAG
FProQ(c)	GTC GCA GGA TAA TCA ACG GA
RProQ(c)	CGT AAT ATC TTC CAC GGC GAA G
FHtpXrec	CATACGATGTGGGTAATCGCATAGTGCGCTTTGTTAAATT GTGTAGGCTGGAGCTGCTTC
RHtpXrec	GCG TCA TTC GAC GCG CTT TTC ATA CTT GCC AGT GGG CTT ACATATGAATATCCTCCTTAG
FHtpX(c)	TTT CTC GTG ACT TAC CGC CT
RHtpX(c)	CGG TAG TGA GCG GTT TAC GTA
Δ*nlpI*-mutant	Rouf SF, 2011, J. Bac

For routine confirmatory PCR, Phusion High-Fidelity PCR master mix with HF buffer was used. The cycling conditions were as follows: 98°C for 1 min and 30 cycles of 98°C for 15 s, annealing temperature for 10 s, 72°C for elongation, and 72°C for 2 min. Annealing temperature varied depending on the primer pairs used, and elongation time was based on the length of the expected product (30 s per kilobase). Oligonucleotide sequences are shown in Table 2.

### Bacteriophage Transduction

Transducing phages (phage P22*int*; Schmieger, 1972) were prepared on strain LB5010 (in LB broth supplemented with D-galactose (Fluka BioChemika) to 0.2% (wt/vol)) or strain 14028s carrying the mutation of interest. The next day chloroform was added to the culture and the culture was vortexed. The culture was then centrifuged for 10 min at 18,500 *g* to create phase separation. The top phase was recovered and used to transfer the genetic marker. The transduction into *S.* Typhimurium SR-11 was done by incubating 20 μl of the P22*int* phage containing the genetic marker with 1ml of exponential phase culture. These were incubated at 37°C with shaking at 220 rpm for 1 h and after washing in PBS plated onto appropriate selective LB agar plates.

### Construction and Isolation of Mutants

The antibiotic marker amplified from either pKD3 for chloramphenicol resistance (*cat* gene) or pKD4 for kanamycin resistance (*neo* gene) was introduced using primers with 3′-ends overlapping the borders of the gene to be deleted, and subsequently inserted into the chromosome; to replace the gene of interest, by double-stranded DNA lambda-red recombination (Datsenko and Wanner, 2000; Yu et al., 2000). As recipients we used *S.* Typhimurium strain LB5010 containing the pSIM6, or *S.* Typhimurium strain 14028 containing pSIM5-tet plasmid, each grown to an OD_600 nm_ of approximately 0.3 at 32°C with shaking at 220 rpm in a water bath. To induce the lambda-red genes, the bacteria were transferred to a 42°C water bath shaking at 220 rpm for 15 min. After cooling on ice for 10 min, bacterial cells were made electrocompetent by washing with ice-cold deionized water four times.

Electroporation of the PCR products generated from pKD3 or pKD4 was done using a Gene Pulser (Bio-Rad, United States) by mixing 25 μl of electrocompetent cells and 0.5 μl of purified PCR product, with settings 1.8 kV, 25 F and 200 Ω. Cells were recovered in 1 ml of LB at 32°C and 220 rpm for 2 h. After recovery the culture was spun down and the pellet was spread on LB agar plates containing either chloramphenicol or kanamycin to select for recombinants. The genetic marker was subsequently transferred to wild-type *S.* Typhimurium SR-11 by phage P22*int* transduction from either the LB5010 or 14028 mutant strains.

Antibiotic markers were removed from the mutants using the plasmid pCP20. Briefly, pCP20 was transferred by P22*int* transduction to the recipient strain containing the antibiotic marker, with selection for colonies on LB agar plates containing ampicillin. Transductants were subcultured three times at 28°C on selective LB agar plates. The bacteria were then subcultured three more times at 37°C to select for loss of plasmid and loss of antibiotic marker. The loss of the antibiotic marker was confirmed by a diagnostic PCR.

Isolation of vancomycin-tolerant Δ*spr*Δ*yebA* mutants was conducted by spreading a LB broth culture of the Δ*spr*Δ*yebA* mutant on a vancomycin gradient TY agar plate. A plate of 14 cm in diameter was poured in a tilted position with 37.5 ml of TY agar containing vancomycin at 40 μg/ml. After solidification, 37.5 ml TY agar lacking antibiotic was poured on top of the solidified TY agar containing vancomycin, now in a horizontal plane. The plate was seeded with about 10^7^ CFU of Δ*spr*Δ*yebA* mutant bacteria in their logarithmic growth phase. After 16 h of incubation yielded colonies were isolated at the higher concentration end of the gradient.

### Plasmid Constructions and Site-Directed Mutagenesis

For creating plasmids for genetic complementation, *spr* and *tsp* were PCR amplified using *S*. Typhimurium SR-11 genomic DNA as template, using oligonucleotide primers to create suitable restriction sites at each end of the amplified fragment. Enzymes used for restriction digestion were SacI or EcoRI, and HindIII (New England Biolabs) while T4 DNA ligase was used to ligate *spr* fragments into vector pBAD30 (Guzman et al., 1995). Following ligation, plasmids were transformed into chemically competent *E. coli* TG1 or Top10 cells (Invitrogen), from which the constructs were purified and electroporated into *S.* Typhimurium LB5010. The plasmids were then transferred into *S.* Typhimurium SR-11 by P22*int* transduction.

### Whole-Genome Sequencing and Analysis

Genomic DNA was prepared from bacterial cultures using a Masterpure DNA Purification Kit (Epicenter). Libraries for sequencing were prepared using Nextera XT sample preparation and index kits (Illumina). The quality of libraries was assessed using a Tapestation 2200 (Agilent) with high-sensitivity D5000 Screentape. Sequencing of the libraries was done using a Miseq device (Illumina) using a 600-cycle V3 reagent kit. The sequences were processed and analyzed using CLC Genomics Workbench 9.0.1 (CLC bio).

### Deoxycholate (DOC) Sensitivity Test

To assay detergent sensitivity, bacteria were diluted 1:100 in LB from an overnight culture, and grown in LB for 2 h at 37°C with shaking at 220 rpm. The OD_600 nm_ of the culture was measured, and the formula [(0.484/OD_600 nm_) × 2.1] was used to adjust the amounts of bacteria to approximately 10^7^ CFU/ml. The bacterial suspension was then diluted 1:100 in distilled water with sodium deoxycholate (DOC; Sigma-Aldrich, Sweden) freshly added to a final concentration of 0.5% (wt/vol). After an incubation of 30 min at room temperature, viable counts were determined from the DOC-suspension by plating dilutions on LB agar plates.

### SDS-PAGE Gel Electrophoresis

Polyacrylamide-bis-acrylamide gel electrophoresis was conducted according to Laemmli (1970), using custom-made 12.5% separation (pH 8.8) and 6% stacking gel (pH 6.8). (Thermo Scientific, Sweden). For solubilization, samples were suspended in reducing protein sample buffer (0.125 M Tris-HCl pH 6.5, 3.6% SDS, 10% β-mercaptoethanol, 2% glycerol, and bromophenol blue) and heated 10 min at 97°C before application on the gel. After completed electrophoresis, gels were stained using Imperial stain.

### Bacterial Membrane Fractionation

The outer membrane fraction was isolated according to Sabet and Schnaitman (1973). Bacteria from overnight LB cultures were diluted 1:100 in TY broth and grown at 37°C for 2 h to mid exponential phase. The OD_600nm_ of the bacterial cultures were measured and then normalized. Bacteria were pelleted by centrifugation at 6,000 *g* for 10 min, re-suspended in PBS, cooled on ice, and disrupted by equal numbers of 10 s sonication pulses until the suspensions visibly cleared. Bacteria were removed by low–speed centrifugation (1,500 *g*), after which the membrane fraction was pelleted by high-speed centrifugation (18,500 *g*, 10 min). The waxy brownish pellet was then re-suspended in 50 mM Tris-HCl buffer containing 10 mM MgCl_2_ and 0.5% Triton X-100. The membrane fraction was again pelleted by centrifugation (18,500 g, 10 min), and the re-suspension and high-speed centrifugation steps were repeated. The final colorless pellet was then suspended in reducing protein sample buffer and run on a 12.5% SDS-PAGE gel as specified above.

### Disk Diffusion Sensitivity Testing

Bacteria were grown overnight in LB broth at 37°C and 220 rpm. The overnight culture was diluted 1:100 in LB broth and grown for 2 h at 37°C and 220 rpm. The OD_600 nm_ was measured of the 2 h culture and the formula [(0.484/OD_600 nm_) × 2.1] was used to estimate the amount of bacteria. An estimated 3 × 10^5^ CFU/ml bacteria were then spread on large (13.7 cm diameter) TY agar plates and the antibiotic disks were placed on top of the bacteria, and the plates were incubated overnight at 37°C. The next day the diameters of the inhibitory zones were measured.

The disks, 6 mm in diameter, were made out of Whatman 3 paper, using an ordinary office paper puncher. Each disk was infused with 5 μl of an antibiotic, and then let to dry. Concentrations of the antibiotics used were; tetracycline 10 μg/ml, vancomycin 20 μg/ml, rifampicin 10 μg/ml, polymyxin B 10 μg/ml, novobiocin 10 μg/ml, and penicillin G 10 μg/ml (Sigma-Aldrich, Sweden). Every antibiotic was dissolved in water except rifampicin, which was dissolved in DMSO.

### MIC Determination

A total amount of 2 × 10^4^ CFU/ml bacteria was prepared and normalized as described above, but using TY broth. This dilution was subsequently pipetted into a 96-well plate, containing TY broth with either vancomycin or penicillin G, resulting in a final concentration of 10^4^ CFU/ml bacteria. The highest concentration for the vancomycin MIC testing was 100 and 50 μg/ml for penicillin G. Then, the antibiotic was diluted down in steps of 1:2 from the highest concentration before an incubation over night at 37°C.

### Drop-on-Lawn

Bacteria were grown overnight in LB broth at 37°C and 220 rpm. The overnight culture was diluted 1:100 in LB broth and grown for 2 h at 37°C and 220 rpm. The OD_600 nm_ was measured of the 2 h culture and the formula [(0.484/OD_600 nm_) × 2.1] was used to calculate the amount of bacteria needed for 10^6^ CFU/ml. From 10^6^ CFU/ml a 1:2 serial dilution series was made in 1ml PBS. From each dilution a 5 μl droplet was pipetted onto TY agar plates containing none, 20 μg/ml, or 40 μg/ml vancomycin, and the TY agar plates were incubated overnight at 37°C. For genetic complementation tests TY agar plates were supplemented with 0.02% (weight/vol) L-arabinose (Sigma-Aldrich, Sweden).

### β-Galactosidase (LacZ) Release Assay

To assay for the release of β-galactosidase (LacZ), pKTH3088-containing strains (KV441–KV448, Table 1) were grown overnight in LB broth at 37°C and 220 rpm. The overnight culture was diluted 1:100 in TY broth and grown for 2 h at 37°C and 220 rpm to mid exponential phase. Following incubation, the OD_600 nm_ of the culture was measured, after which 300 μl of bacteria was added to 700 μl TY broth containing different concentrations of penicillin G or vancomycin and incubated at 37°C for 60 min. When attempting to inhibit penicillin G and vancomycin induced autolysis, 20 μg/ml tetracycline was added during this step. The bacteria were then pelleted by centrifugation and 200 μl of the supernatant was transferred into a tube containing 600 μl reaction buffer [0.001 M MgSO_4_ and 0.05 M β-mercaptoethanol (Sigma-Aldrich, Sweden) in 0.01 M PBS, pH 7.2], and 200 μl of 4 mg/ml ONPG (Sigma-Aldrich, Sweden) dissolved in reaction buffer. The β-galactosidase activity was stopped at given time points by adding 500 μl 0.5 M sodium carbonate. 1ml of the samples were transferred into cuvettes and the β-galactosidase activity was measured using the absorbance at 420 nm as readout. The formula to calculate the arbitrary units (AU) was implemented according to Miller (Miller, 1972).

When measuring proportional or total β-galactosidase activities in bacteria, the samples were similarly incubated overnight and diluted 1:100 the next day. After incubation for 2 h at 37°C, the OD_600 nm_ of the culture was measured and 300 μl culture was added to 700 μl of TY broth containing 40 μg/ml penicillin G and incubated for a further 60 min at 37°C. The samples were pelleted by centrifugation and 200 μl of the supernatant was added to 600 μl of Z-buffer and 200 μl of 4 mg/ml ONPG in reaction buffer. In order to assay the amount of LacZ in the pellet, the remainder of the supernatant was discarded and the pellet resuspended in 100 μl TY broth. This suspension was added to 600 μl of reaction buffer and 200 μl of 4 mg/ml ONPG in reaction buffer supplemented with 50 μl of 0.1% SDS (Sigma-Aldrich, Sweden) and 50 μl of >99% chloroform (Sigma-Aldrich, Sweden) to allow the ONPG to penetrate into the pelleted cells. The samples were then incubated at 30°C for 20 min, sodium carbonate was added, the absorbance at 420 nm measured, and the calculations for the arbitrary units performed as above.

### OD_600 nm_ Determination Following Antibiotic Treatment

As a complement to the β-galactosidase release assay we followed alterations in OD_600 nm_ for the same strains. The strains were grown overnight in LB broth at 37°C and 220 rpm. The overnight culture was diluted 1:100 in TY broth and grown for 2 h at 37°C and 220 rpm to mid-exponential phase. Following this incubation the OD_600 nm_ of the culture was measured to obtain a “pre-antibiotic value.” Simultaneously, 300 μl of bacteria was added to 700 μl TY broth containing different concentrations of vancomycin or penicillin G and incubated at 37°C for 60 min. Following this second incubation the OD_600 nm_ was measured as the “post-antibiotic value.” To quantify the effect of each antibiotic on the OD_600 nm_, the post-antibiotic values were divided with the pre-antibiotic values.

### Viable Bacterial Counts From Broth

Strains were grown overnight in LB broth at 37°C and 220 rpm. The overnight culture was diluted 1:100 in TY broth and grown for 2 h at 37°C and 220 rpm to mid-exponential phase. Following this incubation the viable bacterial count for the input was enumerated by taking 300 μl of bacteria into 700 μl TY broth and a 1:10 serial dilution was performed in PBS. Bacteria were then spread on LB agar plates and incubated overnight at 37°C and the cfu were counted the next day, yielding the input value. In parallel 300 μl of bacteria was added to 700 μl TY broth containing either penicillin G or vancomycin and incubated at 37°C for 60 min. Strains were then serially diluted 1:10 in PBS and the bacteria were spread on LB agar plates, incubated overnight at 37°C, and cfu counted the next day, yielding the output value.

### Statistical Analysis

GraphPad Prism v6.0g (GraphPad Software, Inc., United States) was used for statistical analysis.

## Results

### Lack of Muramyl Endopeptidases Spr, YebA, and YdhO Does Not Result in Growth Defects in *S*. Typhimurium

**FIGURE 1:**
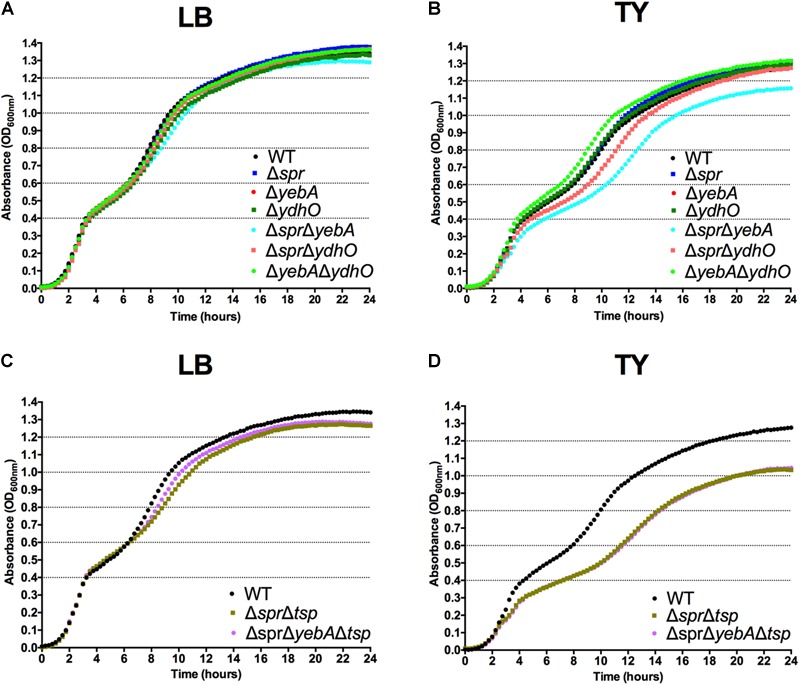
Growth characteristics of *S*. Typhimurium strains. Bacterial strains were diluted to an OD_600 nm_ of 0.01 from overnight LB broth cultures into LB **(A,C)** or TY **(B,D)** broth where after the increase in bacterial density was followed for 24 h.

To assess any functional similarity of the *S*. Typhimurium homologs to the *E. coli* MepS, MepM and MepH proteins regarding growth phenotypes, we constructed single and all combinations of double deletion mutants of *spr, yebA*, and *ydhO* in *S*. Typhimurium SR-11, using allelic replacement (for details, see section “Materials and Methods”). All deletions were verified by PCR. In agreement with observations from *E. coli* (Singh et al., 2012) we were not successful in creating a Δ*spr*Δ*yebA*Δ*ydhO* triple mutant. In agreement with observations made in *E. coli* (Singh et al., 2012) none of the single deletion mutants revealed any significant difference in the overall shape of their growth curves (Figures 1A,B). All single mutants had a similar logarithmic growth rate, and reached a similar optical density in stationary phase in both LB and TY medium. Even the Δ*yebA*Δ*ydhO* mutant grew like the wild-type parental strain. On the other hand, the Δ*spr*Δ*yebA* mutant showed a somewhat decreased rate of replication in TY medium at later stages of the growth slope (Figure 1B). Taken together, these findings suggest a high degree of redundancy for the Spr, YebA, and YhdO endopeptidases in *S*. Typhimurium regarding growth in broth cultures.

### Deletion of Spr Results in Vancomycin Sensitivity Without an Outer Membrane Phenotype

Peptidoglycan turnover and outer membrane synthesis are connected in *E. coli* (Gray et al., 2015), and *E. coli* mutants simultaneously lacking several murein hydrolases show evidence of an outer membrane permeability barrier defect (Heidrich et al., 2002). Therefore, we assessed whether any of the murein endopeptidases Spr, YebA, or YhdO were necessary for maintaining the outer membrane permeability barrier in *S*. Typhimurium. To achieve this, we screened the panel of our *S.* Typhimurium endopeptidase mutants for possible sensitization to six different antibiotics using the disk diffusion method, as well as for detergent tolerance. The antibiotics tested were penicillin G, polymyxin B, tetracycline, rifampicin, novobiocin, and vancomycin. Wild-type *S*. Typhimurium is intrinsically resistant to the latter three due to the outer membrane permeability barrier (Sukupolvi et al., 1984). As comparator strains we used wild-type *S*. Typhimurium SR-11, and three isogenic LPS mutants expected to have an outer membrane permeability defect (Sukupolvi et al., 1984). High salt concentrations has been reported to reduce the sensitivity of *E. coli* to selected antibiotics (Beggs and Andrews, 1976), while low osmolarity would favor the expression of the more permeable outer membrane porin OmpF (Harder et al., 1981; Jaffe et al., 1982) and increase the probability of detecting any sensitization. Thus, sensitivity testing was conducted using low osmolarity TY medium.

As compared to the wild-type, the three LPS mutants, Δ*rfaC* (*waaC*), Δ*rfaG* (*waaG*), and Δ*rfaP* (*waaP*) were each sensitized to polymyxin B, novobiocin, rifampicin, and vancomycin, but not to tetracycline (Table 3). The Δ*spr* mutant was strongly sensitized to vancomycin (inhibition zone increased from 0 to 13 mm), but not to the other antibiotics tested. Subsequent MIC determinations demonstrated that the intrinsic vancomycin resistance was reduced 8-fold for the Δ*spr* mutant and 32-fold for the Δ*spr*Δ*yebA* mutant (Table 3). Also, the Δ*spr*Δ*yebA* mutant revealed a moderate sensitization to novobiocin, penicillin G and rifampicin, while the Δ*yebA* mutant did not show any increase in sensitization to these antibiotics compared to the wild-type (Table 3).

**Table 3:** Antibiotic sensitivity profiles.

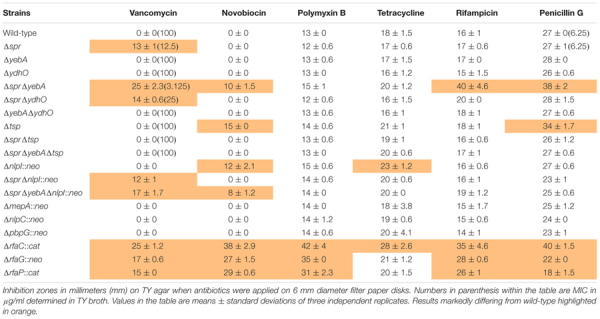

**FIGURE 2:**
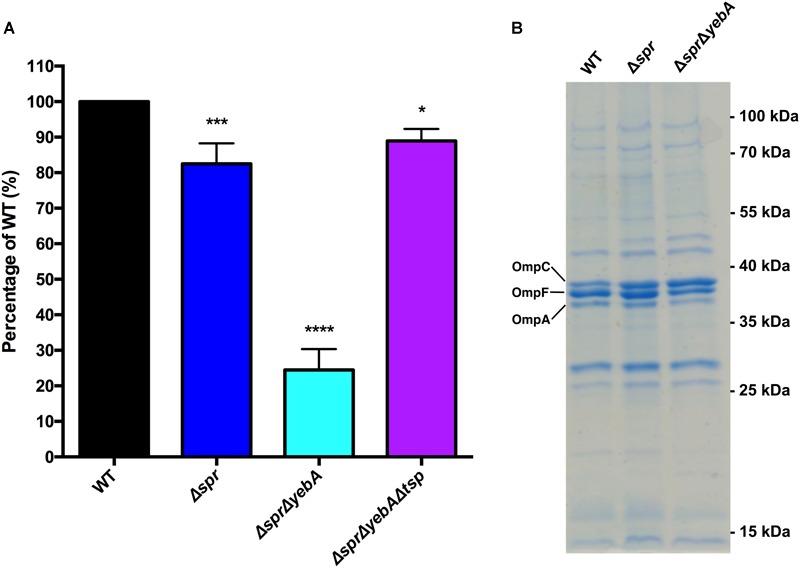
Detergent sensitivity and membrane protein profile of muramyl endopeptidase mutants. **(A)** For probing detergent sensitivity 10^5^ bacteria of each strains was incubated in 0.5% DOC for 30 min, where after the bacterial viable colonies were enumerated. Results are presented as relative CFU yield in relation to the wild-type. The graph shows the mean value and the standard error of the mean of three independent replicates. Statistical test was an one-way ANOVA with Dunnett’s correction for multiple comparisons when comparing the mutants to the wild-type. ^∗^ = p < 0.05, ^∗∗∗^ = p < 0.001, and ^∗∗∗∗^ = p < 0.0001. **(B)** SDS-PAGE gel revealing the outer membrane protein profiles of the wild-type and mutant strains. The positions of pre-stained molecular weight markers are indicated at the right. The labeling of OmpC, OmpF, and OmpA is based on comparison with published data (Sukupolvi et al., 1984; Liu et al., 2016; Choi et al., 2018).

Any sensitization to the detergent deoxycholate (DOC) of the Δ*spr* and Δ*spr*Δ*yebA* mutants was evaluated by incubation in 0.5% DOC for 30 min. In this assay only the Δ*spr*Δ*yebA* mutant showed clear evidence of sensitization (Figure 2A).

### Spr Is the Only Muramyl Endopeptidase to Selectively Maintain Vancomycin Resistance

As the antibiotic sensitization profile caused by the Δ*spr* mutation was unexpected, we next tested whether this mutant phenotype was restricted to the SR-11 line of *S*. Typhimurium. Hence, the Δ*spr* mutation was introduced into the commonly used laboratory *S*. Typhimurium lines SL1344 and 14028. When tested for vancomycin tolerance, the MIC for the wild-type SL1344 and 14028 strains was the same as for the wild-type SR-11 line (100 μg/ml), whereas in the corresponding SL1344 and 14028 Δ*spr* mutant strains the MIC decreased to 12.5 μg/ml, equaling that of the SR-11 Δ*spr* mutant (Table 3).

In *E. coli*, overproduction of PBP7 suppresses thermosensitive growth associated with a *mepS* mutation (Hara et al., 1996), suggesting that PBP7 and MepS connect in parallel pathways. Hence, we deleted *pbpG*, coding for the PBP7 homolog in *S*. Typhimurium. We also created deletion mutants for the *mepA* and *nlpC* homologs in *S*. Typhimurium, each coding for muramyl endopeptidases. Yet, none of the three additional mutants showed sensitization to the antibiotics included in the test panel (Table 3).

In *E. coli*, overexpression of selected outer membrane porin proteins can result in an outer membrane permeability defect (Krishnamoorthy et al., 2016). Yet, outer membrane protein profiles of the wild-type, Δ*spr* and Δ*spr*Δ*yebA* mutants on SDS-PAGE gels did not reveal any significant differences (Figure 2B), arguing that the increased vancomycin sensitization is not caused by a major alteration in outer membrane protein composition.

Our observations combined show that deletion of *spr* in *S*. Typhimurium is associated with sensitization to vancomycin, and that this sensitization is not restricted to line SR-11. Furthermore, we note that the lack of muramyl endopeptidases YebA, YdhO, PBP7, MepA, or NlpC as such do not result in sensitization to vancomycin, nor does the *spr* deletion associate with a general overproduction of major porin proteins.

### Vancomycin Resistance of *S.* Typhimurium Requires a Catalytically Active Spr

**FIGURE 3:**
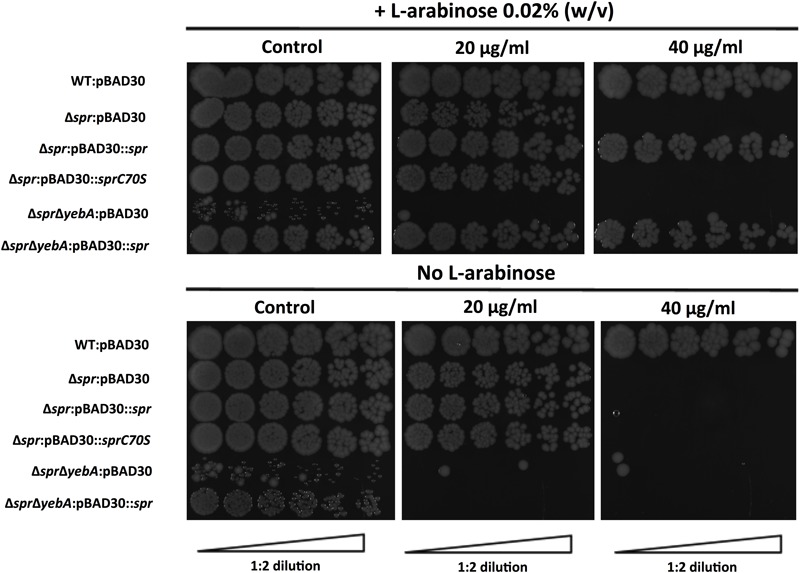
Complementation of vancomycin sensitivity in muramyl endopeptidase mutants as visualized by a drop-on-lawn assay. Wild-type, Δ*spr* and Δ*spr*Δ*yebA* mutants harboring either vector control pBAD30, pBAD30::*spr* or pBAD30::*sprC70S* were serially diluted and spread on TY agar plates, or TY agar plates supplemented with 20 μg/ml or 40 μg/ml of vancomycin, either containing L-arabinose or not. Images are representative of three replicates each.

To further ensure the PCR-based confirmations of the Δ*spr* and Δ*spr*Δ*yebA* mutations, we performed whole genome sequencing on the two mutant constructs, which verified their expected genetic composition. To exclude any potential polar effects of the verified Δ*spr* deletions as a cause of vancomycin sensitization, we applied genetic complementation. We cloned the *spr* gene from *S*. Typhimurium SR-11 under the control of the arabinose-inducible promoter in the plasmid vector pBAD30. There after we generated a point mutation in this plasmid replacing the codon for the conserved catalytic Spr cysteine residue with a codon for serine, creating a C70S alteration in the mature protein (Aramini et al., 2008; Singh et al., 2012). Introducing the cloned native *spr* gene into either the Δ*spr* or Δ*spr*Δ*yebA* mutant fully restored vancomycin resistance, whereas both the empty pBAD30 vector, and the plasmid coding for a catalytically inactive Spr variant, did not restore vancomycin resistance (Figure 3). We conclude that intrinsic vancomycin resistance of *S*. Typhimurium requires a catalytically active Spr.

### Lack of Spr Results in *S.* Typhimurium Being More Prone to Autolyse

Penicillin G activates in *E. coli* a protein-synthesis-dependent autolysis (Prestidge and Pardee, 1957). Hence, we set out to test whether the Δ*spr* mutation would affect any autolytic behavior of *S*. Typhimurium in response to cell wall synthesis inhibitors. To enable quantification of bacterial cell lysis, we adapted a β–galactosidase (LacZ) release assays (see detailed in section “Materials and Methods”). The β–galactosidase release assay is based on the pKTH3088 plasmid (Taira et al., 1991). pKTH3088 carries the *E*. *coli lacZ* gene in the medium copy pACYC184 vector, yielding a constant low yet measurable level of β–galactosidase. This β–galactosidase could be observed in whole cell lysates for all pKTH3088 containing strains, and at equal levels.

**FIGURE 4:**
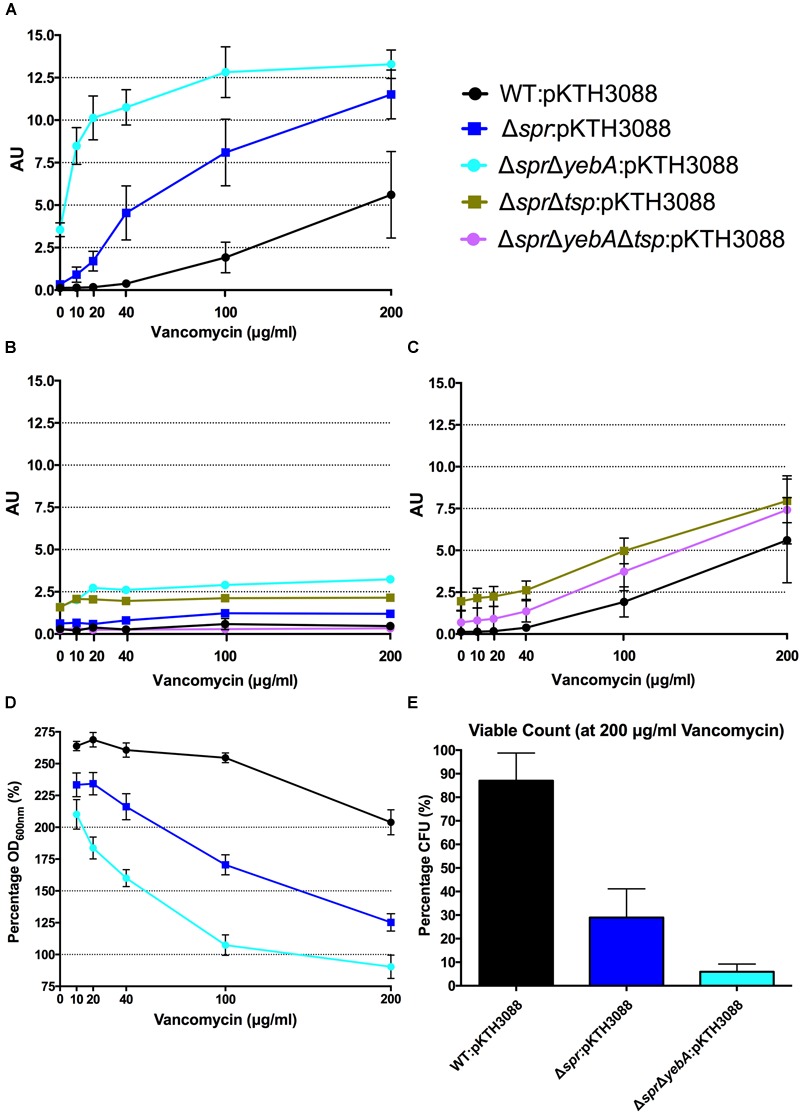
Vancomycin induced autolysis. Release of cytoplasmic β-galactosidase in the presence of increasing concentrations of vancomycin **(A,C)**, or in combination with tetracycline **(B)** after 1 h of incubation at 37^°^C. Decrease in optical density at OD_600_ with increasing vancomycin concentrations **(D)** and viable counts at highest concentration at 200 μg/ml vancomycin after 1 h of incubation **(E)**. Graph presents the mean values and standard error of the mean of three independent replicates.

In this, we incubated a logarithmic growth phase culture of *S*. Typhimurium with increasing concentrations of a cell wall synthesis inhibitor for 1 h, after which the β–galactosidase activities were determined from the culture supernatants. Both the wild-type and Δ*spr* mutant revealed an increased level of β–galactosidase release as a function of increased concentration of vancomycin, with the release being more pronounced for the Δ*spr* mutant (Figure 4A).

To confirm that the vancomycin induced lysis depended on active protein synthesis, we repeated the experiments with tetracycline added into the reaction mixture, as the wild- type and the Δ*spr* mutant exhibited identical MIC:s for tetracycline (Table 3). Adding tetracycline blocked the release of β–galactosidase from both strains (Figure 4B).

**FIGURE 5:**
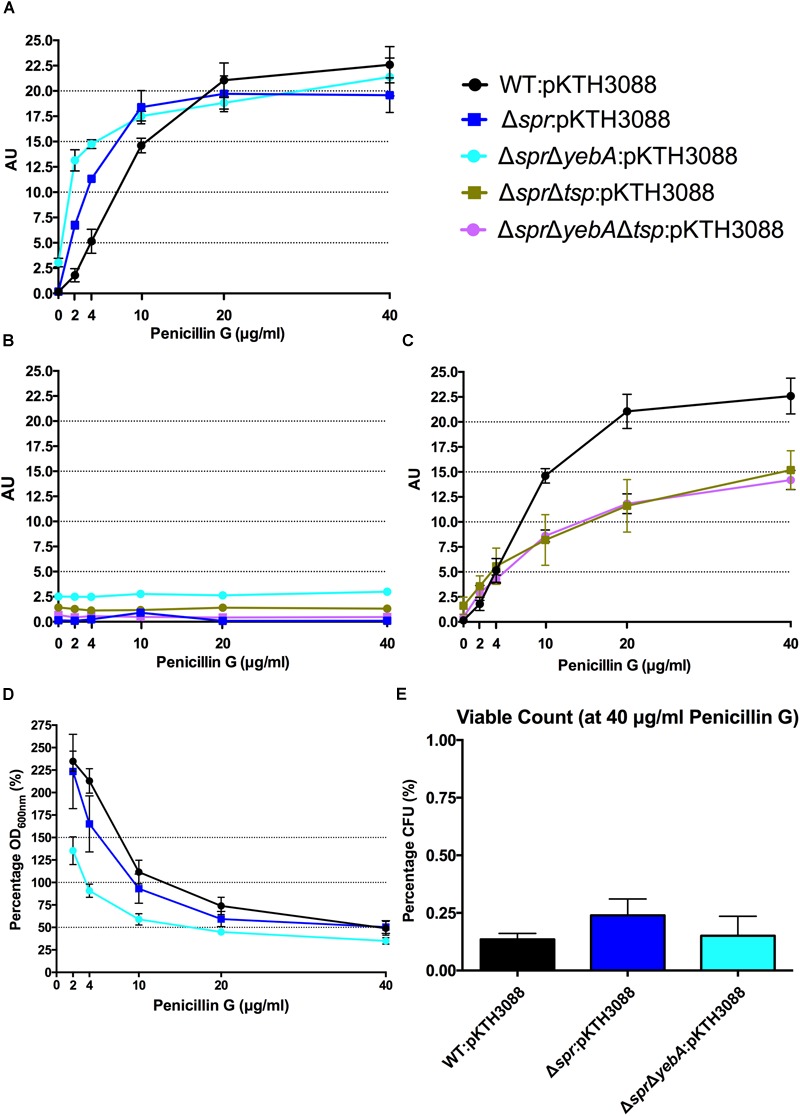
Penicillin G induced autolysis. Release of cytoplasmic β-galactosidase in the presence of increasing concentrations of penicillin G **(A,C)**, or in combination with tetracycline **(B)** after 1 h of incubation at 37°C. Decrease in optical density at OD_600 nm_ with increasing penicillin G concentrations **(D)** and viable counts from highest concentration at 40 μg/ml penicillin G after 1 h of incubation **(E)**. Graph presents the mean values and standard error of the mean of three independent replicates.

As wild-type *S*. Typhimurium SR-11 and the Δ*spr* mutant had an equal MIC for penicillin G (Table 3), we next repeated the lysis assay by replacing vancomycin with penicillin G. Both the wild-type and Δ*spr* mutant reached a similar plateau level of β–galactosidase release at a higher concentration range of penicillin G (Figure 5A). However, compared to the wild-type, the β–galactosidase release was more pronounced in the Δ*spr* mutant at concentrations below the determined 6.25 μg/ml MIC for penicillin G (*t-*test: *p* < 0.01 each for 2 and 4 μg/ml when comparing Δ*spr* mutant to wild-type). Yet again, as for vancomycin, the β–galactosidase release by penicillin G was blocked by addition of tetracycline (Figure 5B).

When we followed the development of the optical density (OD_600 nm_) under the 1 h incubation with cell wall synthesis inhibitors (Figures 4D, 5D), we noted that a substantial proportion of the bacteria apparently remained unlysed. When we determined the viable count from cultures exposed to 200 μg/ml of vancomycin, representing 2-fold MIC for wild-type and 16-fold MIC for Δ*spr* mutant bacteria, we recovered a substantial residual amount of viable bacteria from the cultures (Figure 4E). When the viable counts were measured for the penicillin G exposed cultures (containing antibiotic six times the MIC), we could barely detect any viable bacteria (Figure 5E).

The Δ*spr*Δ*yebA* mutant exhibited a substantial decrease in tolerance to both vancomycin and penicillin G as compared to either the wild-type or Δ*spr* mutant (Table 3). This increased sensitivity was associated with a significantly higher level of β–galactosidase release (relative to the wild-type or the Δ*spr* mutant) after exposure to either antibiotic for 1 h. In combination, these data demonstrate that vancomycin induce a protein-synthesis-dependent autolysis in *S*. Typhimurium, and the intensity of this autolysis inversely correlated with the MIC to vancomycin. On the other hand, penicillin G evoked a more proficient autolysis in the Δ*spr* mutant despite the wild-type and Δ*spr* mutant had the same MIC for penicillin G.

### Periplasmic Protease Tsp Suppresses Δ*spr-*Dependant Vancomycin Sensitivity

**FIGURE 6:**
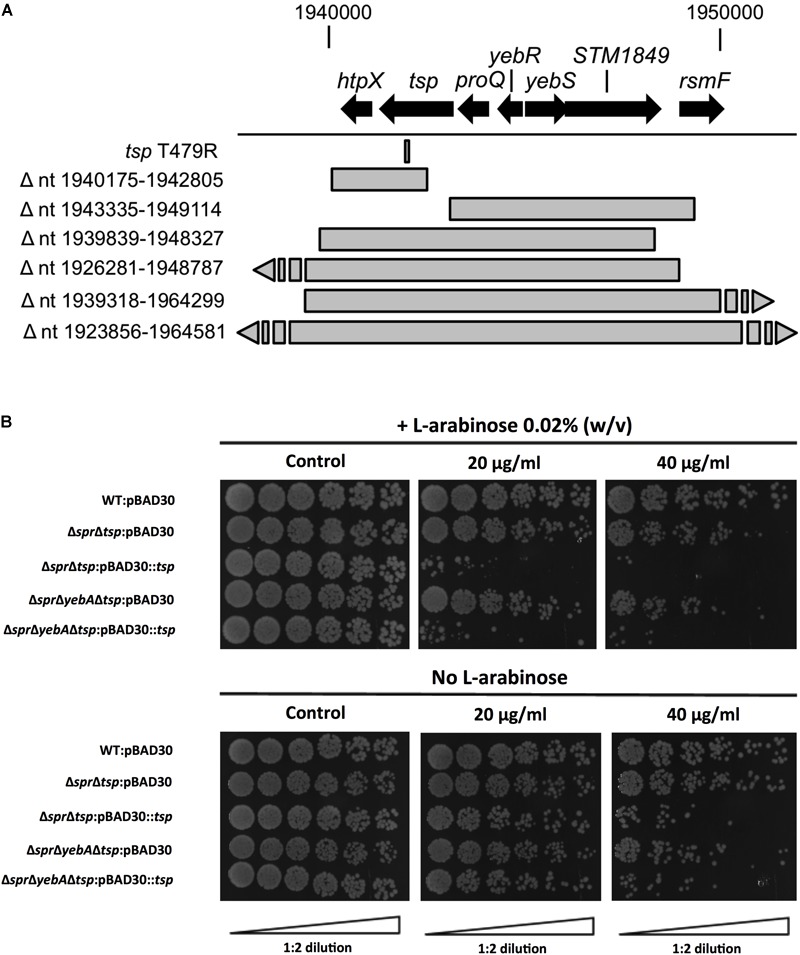
Suppression of vancomycin sensitivity in Δ*spr* mutant backgrounds. **(A)** Genomic visualization of vancomycin tolerant suppressor mutations with the bars representing genomic deletions, except for *tsp* T479R where the bar points to the position of a point mutation. **(B)** Wild-type and the Δ*tsp* suppressed mutants harboring either vector control pBAD30 or full complementation pBAD30::*tsp* were serially diluted and spread on TY agar plates, or TY agar plates supplemented with 20 μg/ml or 40 μg/ml of vancomycin, either containing L-arabinose or not. Images are representative of three replicates each.

Vancomycin-resistant mutants were selected in the Δ*spr*Δ*yebA* background (for details, see section “Materials and Methods”). Twelve vancomycin-tolerant Δ*spr*Δ*yebA* mutants were analyzed by whole genome sequencing. Eleven of the mutants carried overlapping deletions covering nucleotides 1,920,000–1,965,000 in the *S*. Typhimurium LT2 reference genome (Figure 6A). At the center of this region is *tsp*, encoding a periplasmic carboxypeptidase. The remaining suppressor mutant carried a point mutation within *tsp* itself (Figure 6A). These data suggest that inactivation of *tsp* is the common feature of mutations that suppress the vancomycin sensitivity phenotype of the Δ*spr*Δ*yebA* mutant.

To confirm the *tsp* mutations as suppressors, we deleted *tsp*, as well as the individual genes that mapped upstream and downstream of *tsp* (*proQ* and *htpX*) in the Δ*spr*Δ*yebA* mutant. Out of these three constructs, only deletion of *tsp* resulted in a vancomycin resistant phenotype in the Δ*spr*Δ*yebA* mutant (Table 3). In addition, when the *tsp* deletion was introduced into the Δ*spr* mutant, it converted the phenotype from vancomycin sensitive to vancomycin resistant (Table 3). Conversely, when a cloned *tsp* gene was introduced into the vancomycin resistant Δ*spr*Δ*tsp* and Δ*spr*Δ*yebA*Δ*tsp* mutants, the phenotypes were reverted to vancomycin sensitive (Figure 6B).

The Δ*tsp* mutation also reverted the general antibiotic sensitization of the Δ*spr*Δ*yebA* mutant without affecting growth (Table 3, Figures 1C,D). In addition, introduction of the *tsp* deletion into the Δ*spr* and Δ*spr*Δ*yebA* mutants suppressed the autolysis by reducing their release of β–galactosidase in the presence of vancomycin or penicillin G (Figures 4C, 5C). Thus, a *tsp* deletion acted as a general suppressor mutation for Δ*spr* and Δ*spr*Δ*yebA* mutant phenotypes. That said, the *tsp* deletion alone did not confer increased vancomycin resistance (Table 3).

In *E. coli*, Tsp co-purifies with the outer membrane lipoprotein NlpI, which assists Tsp in degrading MepS (Spr) (Singh et al., 2015). Furthermore, *nlpI* mutations suppress a temperature-sensitive phenotype of an *E. coli*Δ*mepS* mutant (Tadokoro et al., 2004) implying a functional connection between Tsp, NlpI and MepS. Yet, when we deleted *nlpI* in the *S*. Typhimurium Δ*spr* and Δ*spr*Δ*yebA* mutants, they retained their sensitization to vancomycin (Table 3). Hence the vancomycin-sensitive phenotype of the Δ*spr* mutant is mainly dependent on Tsp rather than on NlpI.

## Discussion

In *E. coli*, mutants that lack any one of the muramyl endopeptidases, Spr (MepS), YebA (MepM) or YhdO (MepH), suffer no obvious growth defects (Singh et al., 2012). In agreement with this, we found that genetic deletion of the individual murein endopeptidases, Spr, YebA, or YhdO in *S*. Typhimurium did not affect bacterial growth in broth cultures. We also created Δ*spr*, Δ*yebA*, and Δ*yhdO* double mutants, to test for redundancy in their contribution to *S*. Typhimurium growth in broth. Only for the Δ*spr*Δ*yebA* mutant, did we note a minor defect in growth capacity. This phenotype was not seen with any of the single mutants, or with the Δ*yebA*Δ*yhd*O double mutant, suggesting that *spr* might not be completely non-redundant under the growth conditions tested.

In Gram-negative enteric bacteria, the outer membrane acts as a barrier adding to intrinsic resistance to lysozyme, and to antibiotics such as novobiocin, rifampicin and vancomycin (Grundström et al., 1980; Helander et al., 1989). Lipopolysaccharide (LPS) contributes to outer membrane integrity and mutations in genes involved in LPS biosynthesis can sensitize Gram-negative bacteria simultaneous to numerous antibiotics (Grundström et al., 1980; Helander et al., 1989). Outer membrane integrity can also be disturbed by the expression of aberrant outer membrane proteins (Rhen et al., 1988) or polymyxin B nonapeptide (Vaara and Vaara, 1983; Ofek et al., 1994). Simultaneous genetic depletion of multiple murein hydrolases may also cause outer membrane destabilization (Heidrich et al., 2002) in *E. coli*, including vancomycin sensitization (Korsak et al., 2005).

Here we report that depletion of a single muramyl endopeptidase alone, Spr, results in vancomycin sensitization in an enteric bacterium. Significantly, the vancomycin sensitization associated with the Δ*spr* mutation did not associate with sensitization to rifampicin or novobiocin, which would be expected in the case of classical outer membrane destabilization. Recent work on *Vibrio cholerae* suggested that mechanisms other than outer membrane permeability are also involved in preventing antibiotics from entering, or acting, in the periplasm (Dörr et al., 2016). Our observation that the growth of wild-type *S*. Typhimurium is inhibited by vancomycin, albeit at a high concentration (Table 3), suggests that also in *S*. Typhimurium the outer membrane barrier does not completely prevent vancomycin entry. Consequently, at high concentrations vancomycin could accumulate to a level that prevents efficient peptidoglycan cross-linking. In the Δ*spr* mutant the capacity to ensure peptidoglycan turnover, while not yet preventing growth, could be compromised as such. In the Δ*spr* mutant vancomycin concentrations sub-inhibitory for wild-type bacteria would further add to disturbed peptidoglycan composition and consequently lower the threshold for preventing growth.

In *E. coli*, blocking peptidoglycan cross-linking with penicillin G results in autolysis. Thus we argued that increased vancomycin sensitivity of the Δ*spr* mutant could associate with an altered autolytic behavior. Hence, we set out to compare the autolytic behavior of wild-type and Δ*spr* mutants by measuring release of the cytoplasmic enzyme β–galactosidase after vancomycin exposure. Release of β–galactosidase was more prominent for the Δ*spr* mutant, and notably so the for the Δ*spr*Δ*yebA* mutant (Figure 4A). Thus, vancomycin induced an autolysis in *S*. Typhimurium that inversely correlated with the vancomycin MIC. To demonstrate that the increased autolysis of the Δ*spr* mutant was not only a reflection of a decreased MIC for vancomycin, we repeated the autolysis assay upon exposure to penicillin G, for which the wild-type and *spr* mutant expressed an equal MIC (Table 3). In this, the Δ*spr* mutant revealed a more rapid onset of autolysis upon exposure to penicillin G (Figure 5A).

When recording autolysis measured as decrease in optical density, we noted that a proportion of each culture exposed to either vancomycin or penicillin G remained apparently non-lysed (Figures 4D, 5D). Even at a vancomycin concentration that was 16-fold MIC, we recovered a substantial proportion of viable Δ*spr* bacteria at the end of the experiment (Figure 4E). Viable bacteria were also recovered from the corresponding penicillin G-exposed cultures but at 100-fold lower frequency for both wild-type and Δ*spr* mutant bacteria (Figure 5E). This would imply that the Δ*spr* mutant indeed is more prone to autolysis, and that the PBPs of wild-type and Δ*spr* mutant bacteria, whether autolytic or not, are equally and irreversibly inhibited by penicillin G.

In Gram-positive bacteria vancomycin resistance is achieved through the acquisition of large genetic blocks coding for new peptidoglycan motifs, rather than through point mutations (Gardete and Tomasz, 2014; Faron et al., 2016). As the MIC for our vancomycin-sensitive Δ*spr*Δ*yebA* mutant under our test conditions approached MIC values of susceptible Gram-positive pathogens such as *Enterococcus faecalis* and *Staphylococcus aureus* [Susceptible Enterococci spp. ≤4 μg/ml; susceptible^1^ *S. aureus* ≤ 2 μg/ml, “EUCAST: Clinical Breakpoints”, 2018], we set out to probe whether we could in a Gram-negative vancomycin-sensitive bacterium select for spontaneous mutations contributing to vancomycin tolerance. In this, we selected and genetically confirmed that Δ*spr*-mediated vancomycin sensitization, including the more pronounced sensitization of the Δ*spr*Δ*yebA* mutant, required a *tsp*-proficient genetic background (Figure 6). While corresponding Spr or Tsp proteins do not exist in enterococci, the success in isolating vancomycin-resistant mutations, including point mutations, in our Δ*spr*Δ*yebA* mutant background adds to our understanding of how vancomycin resistance in a sensitized genetic background can be achieved without horizontal gene transfer. We here also demonstrate that intrinsic *S*. Typhimurium vancomycin resistance relies on a catalytically active Spr (Figure 3). This, and the fact that the vancomycin sensitivities of the Δ*spr* and Δ*spr*Δ*yebA* mutants are close to the clinical breakpoints of relevant clinical Gram-positive pathogens places the catalytic activity of Spr and related endopeptidases as potential target whose inhibition could potentiate treatment of enteric bacterial infections with vancomycin.

Tsp is a periplasmic endopeptidase implicated in the processing of Spr and PBP3 (Hara et al., 1989, 1991; Singh et al., 2015). In *Pseudomonas aeruginosa* the YebA/MepM homolog is also subjected to proteolysis (Srivastava et al., 2018). Thus, there might exist analogous protein complexes in a wide range of bacteria that regulate turnover of peptidoglycan. Depletion of any component of such a complex could distort the cell wall composition with accompanying sensitization to antibiotics. Indeed, in *S*. Typhimurium the *tsp* mutant revealed sensitization to novobiocin and penicillin, a phenotype suppressed by deleting *spr* (Table 3). Even so, the *tsp* mutant exhibited the same MIC for vancomycin as the wild-type strain (Table 3).

In summary, we have genetically defined a new pathway for intrinsic resistance to the large-molecular-weight antibiotic vancomycin that is not dependent on outer membrane permeability, in the Gram-negative pathogen *S*. Typhimurium. This new pathway involves the combined action of the muramyl endopeptidase Spr, together with the protease Tsp, in maintaining the peptidoglycan homeostasis essential for maintaining the cell wall integrity of the bacterium upon antibiotic challenge. These insights add to the knowledge needed to combat the increasing problem of antibiotic resistance in Gram-negative bacteria.

## Author Contributions

KV, HW, DH, and MR designed the study. KV, DLH, IS, and MR performed the experiments. KV, DH, and MR wrote the manuscript.

## Conflict of Interest Statement

The authors declare that the research was conducted in the absence of any commercial or financial relationships that could be construed as a potential conflict of interest.

## Footnotes

1 http://www.eucast.org
