# Corrigendum: Muramyl Endopeptidase Spr Contributes to Intrinsic Vancomycin Resistance in *Salmonella enterica* Serovar Typhimurium

**DOI:** 10.3389/fmicb.2019.00386

**Published:** 2019-03-05

**Authors:** Kim Vestö, Douglas L. Huseby, Iina Snygg, Helen Wang, Diarmaid Hughes, Mikael Rhen

**Affiliations:** ^1^Department of Microbiology, Tumor and Cell Biology, Karolinska Institutet, Stockholm, Sweden; ^2^Department of Medical Biochemistry and Microbiology, Uppsala University, Uppsala, Sweden; ^3^Laboratory for Molecular Infection Medicine Sweden (MIMS), Department of Molecular Biology, Umeå Centre for Microbial Research (UCMR), Umeå University, Umeå, Sweden

**Keywords:** vancomycin, antibiotic resistance, Spr, MepS, YebA, MepM, Tsp, Prc

In the original article, there was an error. We stated that “A similar phenotype was not achieved by deleting the genes coding for muramyl endopeptidase MepA, PbpG, NlpC, YedA, or YhdO”. This is incorrect as the correct names for “YedA” is “YebA” and the correct name for “YhdO” is “YdhO.”

A correction has been made to the **Abstract**:

“The impermeability barrier provided by the outer membrane of enteric bacteria, a feature lacking in Gram-positive bacteria, plays a major role in maintaining resistance to numerous antimicrobial compounds and antibiotics. Here we demonstrate that mutational inactivation of *spr*, coding for a muramyl endopeptidase, significantly sensitizes *Salmonella enterica* serovar Typhimurium to vancomycin without any accompanying apparent growth defect or outer membrane destabilization. A similar phenotype was not achieved by deleting the genes coding for muramyl endopeptidases MepA, PbpG, NlpC, YebA, or YdhO. The *spr* mutant showed increased autolytic behavior in response to not only vancomycin, but also to penicillin G, an antibiotic for which the mutant displayed a wild-type MIC. A screen for suppressor mutations of the *spr* mutant phenotype revealed that deletion of *tsp (prc)*, encoding a periplasmic carboxypeptidase involved in processing Spr and PBP3, restored intrinsic resistance to vancomycin and reversed the autolytic phenotype of the spr mutant. Our data suggest that Spr contributes to intrinsic antibiotic resistance in *S*. Typhimurium without directly affecting the outer membrane permeability barrier. Furthermore, our data suggests that compounds targeting specific cell wall endopeptidases might have the potential to expand the activity spectrum of traditional Gram-positive antibiotics.”

The authors apologize for this error and state that this does not change the scientific conclusions of the article in any way.

